# 7-Meth­oxy-2-phenyl­quinoline-3-carbaldehyde

**DOI:** 10.1107/S1600536814001457

**Published:** 2014-01-22

**Authors:** Hasna Hayour, Abdelmalek Bouraiou, Sofiane Bouacida, Saida Benzerka, Ali Belfaitah

**Affiliations:** a Laboratoire des Produits Naturels d’Origine Végétale et de Synthèse Organique, PHYSYNOR Université Constantine, 25000 Constantine, Algeria; b Unité de Recherche de Chimie de l’Environnement et Moléculaire Structurale, CHEMS, Université Constantine, 25000 , Algeria; c Département Sciences de la Matière, Faculté des Sciences Exactes et Sciences de la Nature et de la Vie, Université , Oum El Bouaghi, 04000 Oum El Bouaghi, Algeria

## Abstract

In the title mol­ecule, C_17_H_13_NO_2_, the phenyl ring is inclined to the quinoline ring system by 43.53 (4)°. In the crystal, mol­ecules are linked *via* C—H⋯O hydrogen bonds, forming double-stranded chains propagating along [011]. These chains are linked *via* π–π inter­actions involving inversion-related quinoline rings; the shortest centroid–centroid distance is 3.6596 (17) Å.

## Related literature   

For the synthesis and applications of similar structures, see: Montalban (2011 ▶); Wang *et al.* (2011 ▶); Nilsson *et al.* (2008 ▶); Elliott *et al.* (2006 ▶); Metallidis *et al.* (2007 ▶); Kaila *et al.* (2007 ▶). For related structures, see: Abdel-Wahab *et al.* (2012 ▶); Benzerka *et al.* (2011 ▶, 2012 ▶, 2013 ▶). For our previously work on the imidazol unit, see: Bouraiou *et al.* (2011 ▶); Hayour *et al.* (2011 ▶); Benzerka *et al.* (2012 ▶).
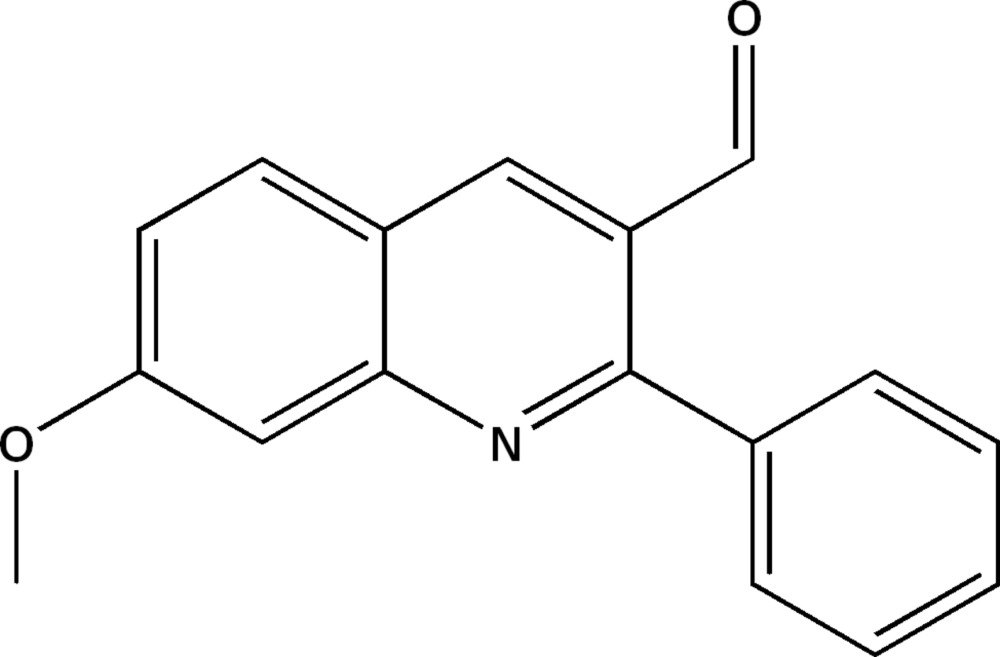


## Experimental   

### 

#### Crystal data  

C_17_H_13_NO_2_
*M* *_r_* = 263.28
Triclinic, 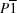

*a* = 7.332 (3) Å
*b* = 7.582 (2) Å
*c* = 12.487 (4) Å
α = 73.424 (12)°
β = 85.877 (12)°
γ = 83.029 (11)°
*V* = 659.9 (4) Å^3^
*Z* = 2
Mo *K*α radiation
μ = 0.09 mm^−1^
*T* = 150 K
0.12 × 0.03 × 0.02 mm

#### Data collection  

Bruker APEXII diffractometer
Absorption correction: multi-scan (*SADABS*; Sheldrick, 2002 ▶) *T*_min_ = 0.889, *T*_max_ = 0.993
5538 measured reflections
3001 independent reflections
2344 reflections with *I* > 2σ(*I*)
*R*_int_ = 0.044

#### Refinement  

*R*[*F* ^2^ > 2σ(*F* ^2^)] = 0.048
*wR*(*F* ^2^) = 0.138
*S* = 1.06
3001 reflections
182 parameters
H-atom parameters constrained
Δρ_max_ = 0.23 e Å^−3^
Δρ_min_ = −0.24 e Å^−3^

### 

Data collection: *APEX2* (Bruker, 2001 ▶); cell refinement: *SAINT* (Bruker, 2001 ▶); data reduction: *SAINT*; program(s) used to solve structure: *SIR2002* (Burla *et al.*, 2005 ▶); program(s) used to refine structure: *SHELXL97* (Sheldrick, 2008 ▶); molecular graphics: *ORTEP-3 for Windows* (Farrugia, 2012 ▶) and *DIAMOND* (Brandenburg & Berndt, 2001 ▶); software used to prepare material for publication: *WinGX* publication routines (Farrugia, 2012 ▶) and *CRYSCAL* (T. Roisnel, local program).

## Supplementary Material

Crystal structure: contains datablock(s) I. DOI: 10.1107/S1600536814001457/hg5376sup1.cif

Structure factors: contains datablock(s) I. DOI: 10.1107/S1600536814001457/hg5376Isup2.hkl

Supporting information file. DOI: 10.1107/S1600536814001457/hg5376Isup3.cml

CCDC reference:

Additional supporting information:  crystallographic information; 3D view; checkCIF report

## Figures and Tables

**Table 1:** Hydrogen-bond geometry (Å, °)

*D*—H⋯*A*	*D*—H	H⋯*A*	*D*⋯*A*	*D*—H⋯*A*
C5—H5⋯O2^i^	0.95	2.48	3.349 (2)	153
C15—H15⋯O1^ii^	0.95	2.54	3.377 (2)	148

Symmetry codes: (i) 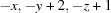; (ii) 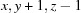.

**Fig. 1.**
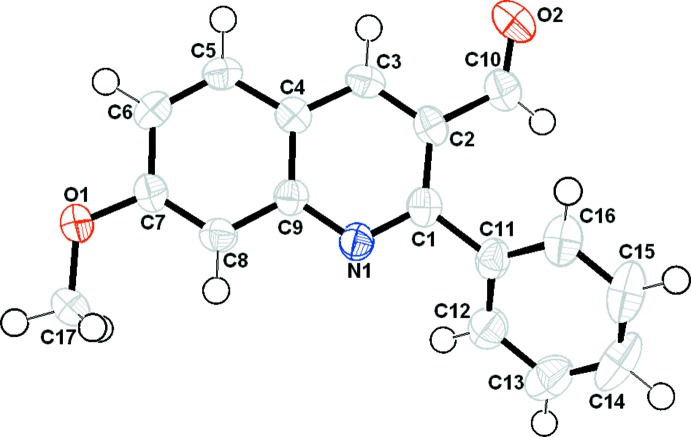
(Farrugia, 2012) The molecular geometry of (I) with the atom-labelling scheme. Displacement ellipsoids are drawn at the 50% probability level. H atoms are represented as small spheres of arbitrary radius.

**Fig. 2.**
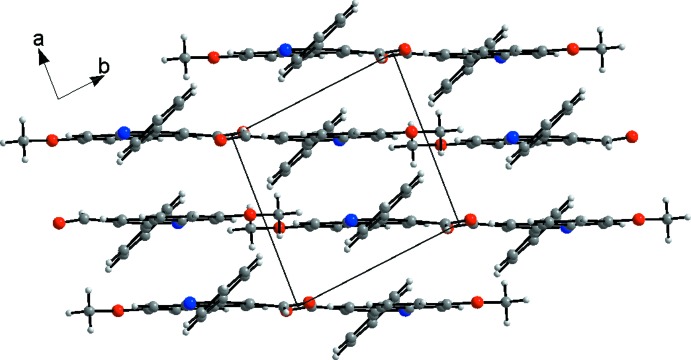
(Brandenburg & Berndt, 2001)A alternating layers parallel to (210)planes of (I) viewed down the c axis.

**Fig. 3.**
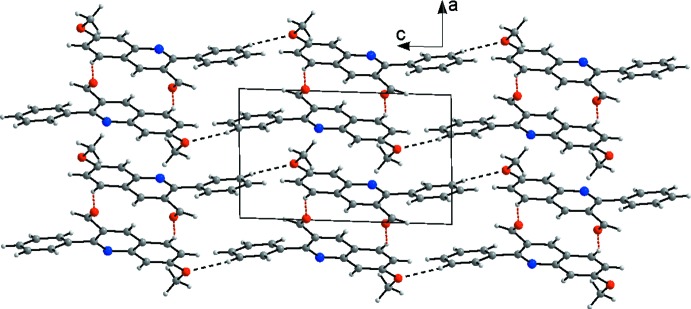
(Brandenburg & Berndt, 2001) A diagram of the layered crystal packing of (I) viewed down the b axis showing hydrogen bond as dashed line.

## supplementary crystallographic information

### 1. Comment

Heterocyclic compounds have so far been synthesized mainly due to the wide range of biological activities. Quinoline derivatives have considerable interest since many years due to the presence of this skeleton in a large number of bioactive compounds and natural products (Montalban, 2011; Wang *et al.*, 2011). In other hand, it has been well established that presence of aryl ring at second position of quinoline moiety gives a very good antibacterial property to the target molecule and plays a significant role in development of new antibacterials (Nilsson *et al.*, 2008; Elliott *et al.*, 2006). These derivatives were found to be useful biological targets, and at present they attained much attention in the development of new drugs (Metallidis *et al.*, 2007; Kaila *et al.*, 2007). Following of our previous works related to the use of substituted 2-chloro-3-formylquinolines as precursors of different quinoline-containing heterocycles (Bouraiou *et al.*, 2011; Hayour *et al.*, 2011), we have recently reported preparations and antibacterial screening of series of compounds carrying diverse functionalities such as an amine, amide, ester group, heterocylic unit linked to the 2-phenylquinoline entity (Benzerka *et al.*, 2011, 2012, 2013). We report herein the synthesis and single-crystal X-ray structure of 7-methoxy-2-phenylquinoline-3-carbaldehyde (I).

The molecular geometry and the atom-numbering scheme of (I) are shown in Fig. 1. The asymmetric unit of (I) consists of 2-phenylquinoline linked to 7-methoxy and 3-carbaldehyde. The substituted phenyl ring forms dihedral angle of 43.53 (4)° with heterocyclic ring of quinoline. The crystal packing can be described as alternating layers parallel to the (210) (Fig. 2). It is stabilized by C—H···O hydrogen bond (Fig. 3; Table. 1), and strong π-π stacking interactions between quinoline rings with a centroid-centroid distance from 3.6596 (17)Å to 4.0726 (18)Å. These interaction bonds link the molecules within the layers and also link the layers together, reinforcing the cohesion of the structure.

### 2. Experimental

A mixture of 2-chloro-7-methoxyquinoline-3-carbaldehyde (l mmol) and phenylboronic acid (1.2 mmol) in 4 ml DME was stirred under nitrogen. Palladium acetate (0.01 mmol), aq. K_2_CO_3_ (3 mmol in 3.75 ml of H_2_O) and triphenylphosphine (0.04 mmol) were added and the mixture was refluxed for 2 h. After completion, the reaction mixture was cooled to room temperature, diluting with EtOAc and filtering through a small bed of celite. The organic layers were collected, combined, washed with water and saturated aq NaHCO_3_ (2x10 ml), dried over anhydrous Na_2_SO_4_ and concentrated under vacuum. The crude compound was purified by column chromatography on silica gel using ethyl acetate/ hexane (1/2) to afford the desired product as yellow solid. Single crystals suitable for the X-ray diffraction analysis were obtained by dissolving the pure compound in an ethyl acetate/hexane mixture and allowing the solution to slowly evaporate at room temperature.

### 3. Refinement

. Approximate positions for all the H atoms were first obtained from the difference electron density map. However, the H atoms were situated into idealized positions and the H-atoms have been refined within the riding atom approximation. The applied constraints were as follow: C_aryl_—H_aryl_ = 0.95 Å; and C_methyl_—H_methyl_ = 0.98 Å; The idealized methyl group was allowed to rotate about the C—C bond during the refinement by application of the command AFIX 137 in *SHELXL97* (Sheldrick, 2008). *U*_iso_(H_methyl_) = 1.5*U*_eq_(C_methyl_) or *U*_iso_(H_aryl_) = 1.2 *U*_eq_(C_aryl_).

### Crystal data

**Table:**

C_17_H_13_NO_2_	*Z* = 2
*M**_r_* = 263.28	*F*(000) = 276
Triclinic, *P*1	*D*_x_ = 1.325 Mg m^−^^3^
*a* = 7.332 (3) Å	Mo *K*α radiation, λ = 0.71073 Å
*b* = 7.582 (2) Å	Cell parameters from 1596 reflections
*c* = 12.487 (4) Å	θ = 2.9–27.7°
α = 73.424 (12)°	µ = 0.09 mm^−^^1^
β = 85.877 (12)°	*T* = 150 K
γ = 83.029 (11)°	Stick, yellow
*V* = 659.9 (4) Å^3^	0.12 × 0.03 × 0.02 mm

### Data collection

**Table:**

Bruker APEXII diffractometer	2344 reflections with *I* > 2σ(*I*)
Graphite monochromator	*R*_int_ = 0.044
CCD rotation images, thin slices scans	θ_max_ = 27.7°, θ_min_ = 2.8°
Absorption correction: multi-scan (*SADABS*; Sheldrick, 2002)	*h* = −9→9
*T*_min_ = 0.889, *T*_max_ = 0.993	*k* = −9→9
5538 measured reflections	*l* = −15→16
3001 independent reflections	

### Refinement

**Table:**

Refinement on *F*^2^	Primary atom site location: structure-invariant direct methods
Least-squares matrix: full	Secondary atom site location: difference Fourier map
*R*[*F*^2^ > 2σ(*F*^2^)] = 0.048	Hydrogen site location: inferred from neighbouring sites
*wR*(*F*^2^) = 0.138	H-atom parameters constrained
*S* = 1.06	*w* = 1/[σ^2^(*F*_o_^2^) + (0.064*P*)^2^ + 0.0518*P*] where *P* = (*F*_o_^2^ + 2*F*_c_^2^)/3
3001 reflections	(Δ/σ)_max_ < 0.001
182 parameters	Δρ_max_ = 0.23 e Å^−^^3^
0 restraints	Δρ_min_ = −0.24 e Å^−^^3^

### Special details

**Table:**

Geometry. All e.s.d.'s (except the e.s.d. in the dihedral angle between two l.s. planes) are estimated using the full covariance matrix. The cell e.s.d.'s are taken into account individually in the estimation of e.s.d.'s in distances, angles and torsion angles; correlations between e.s.d.'s in cell parameters are only used when they are defined by crystal symmetry. An approximate (isotropic) treatment of cell e.s.d.'s is used for estimating e.s.d.'s involving l.s. planes.
Refinement. Refinement of *F*^2^ against ALL reflections. The weighted *R*-factor *wR* and goodness of fit *S* are based on *F*^2^, conventional *R*-factors *R* are based on *F*, with *F* set to zero for negative *F*^2^. The threshold expression of *F*^2^ > σ(*F*^2^) is used only for calculating *R*-factors(gt) *etc*. and is not relevant to the choice of reflections for refinement. *R*-factors based on *F*^2^ are statistically about twice as large as those based on *F*, and *R*- factors based on ALL data will be even larger.

### Fractional atomic coordinates and isotropic or equivalent isotropic displacement parameters (Å^2^)

**Table:**

	*x*	*y*	*z*	*U*_iso_*/*U*_eq_	
C17	0.5020 (2)	−0.08593 (18)	0.69768 (12)	0.0260 (3)	
H17A	0.4055	−0.1161	0.6575	0.039*	
H17B	0.5425	−0.1947	0.7585	0.039*	
H17C	0.6065	−0.0489	0.6459	0.039*	
C1	0.21611 (18)	0.61093 (18)	0.31036 (11)	0.0201 (3)	
C2	0.13397 (18)	0.75805 (18)	0.35386 (11)	0.0204 (3)	
C3	0.13446 (18)	0.73428 (18)	0.46777 (12)	0.0204 (3)	
H3	0.0814	0.831	0.4983	0.025*	
C4	0.21293 (17)	0.56801 (17)	0.53832 (11)	0.0187 (3)	
C5	0.21827 (18)	0.53151 (18)	0.65648 (11)	0.0208 (3)	
H5	0.1691	0.6246	0.691	0.025*	
C6	0.29264 (19)	0.36550 (19)	0.72048 (11)	0.0221 (3)	
H6	0.2969	0.3434	0.7991	0.026*	
C7	0.36406 (18)	0.22478 (18)	0.66966 (11)	0.0202 (3)	
C8	0.36215 (18)	0.25374 (18)	0.55626 (11)	0.0203 (3)	
H8	0.4115	0.1585	0.5236	0.024*	
C9	0.28617 (17)	0.42649 (17)	0.48809 (11)	0.0184 (3)	
C10	0.03347 (19)	0.92782 (18)	0.28371 (12)	0.0249 (3)	
H10	0.0004	0.9279	0.2115	0.03*	
C11	0.23061 (18)	0.62767 (19)	0.18804 (11)	0.0222 (3)	
C12	0.19420 (19)	0.4795 (2)	0.15046 (12)	0.0262 (3)	
H12	0.1563	0.3708	0.2025	0.031*	
C13	0.2129 (2)	0.4898 (2)	0.03792 (13)	0.0346 (4)	
H13	0.1891	0.3876	0.0133	0.042*	
C14	0.2660 (2)	0.6478 (3)	−0.03901 (13)	0.0426 (4)	
H14	0.2765	0.6549	−0.1164	0.051*	
C15	0.3037 (2)	0.7956 (2)	−0.00300 (13)	0.0399 (4)	
H15	0.3419	0.9035	−0.0556	0.048*	
C16	0.2860 (2)	0.7864 (2)	0.10982 (12)	0.0302 (4)	
H16	0.3115	0.8885	0.1341	0.036*	
N1	0.28755 (15)	0.45003 (15)	0.37525 (9)	0.0202 (3)	
O1	0.43121 (14)	0.06304 (12)	0.74313 (8)	0.0250 (3)	
O2	−0.00907 (16)	1.06677 (13)	0.31310 (9)	0.0343 (3)	

### Atomic displacement parameters (Å^2^)

**Table:**

	*U*^11^	*U*^22^	*U*^33^	*U*^12^	*U*^13^	*U*^23^
C17	0.0318 (8)	0.0184 (7)	0.0253 (7)	0.0039 (6)	−0.0022 (6)	−0.0045 (6)
C1	0.0174 (6)	0.0220 (7)	0.0197 (7)	−0.0042 (5)	0.0000 (5)	−0.0032 (5)
C2	0.0184 (7)	0.0177 (7)	0.0227 (7)	−0.0025 (5)	−0.0008 (5)	−0.0016 (5)
C3	0.0195 (7)	0.0178 (6)	0.0247 (7)	−0.0012 (5)	0.0015 (5)	−0.0079 (5)
C4	0.0162 (6)	0.0188 (7)	0.0209 (7)	−0.0031 (5)	0.0004 (5)	−0.0049 (5)
C5	0.0216 (7)	0.0209 (7)	0.0216 (7)	−0.0021 (5)	0.0021 (5)	−0.0097 (5)
C6	0.0232 (7)	0.0267 (7)	0.0175 (6)	−0.0034 (6)	0.0002 (5)	−0.0079 (5)
C7	0.0203 (7)	0.0186 (7)	0.0196 (7)	−0.0014 (5)	−0.0012 (5)	−0.0023 (5)
C8	0.0213 (7)	0.0188 (7)	0.0209 (7)	−0.0001 (5)	0.0010 (5)	−0.0069 (5)
C9	0.0171 (6)	0.0194 (7)	0.0180 (6)	−0.0029 (5)	0.0006 (5)	−0.0043 (5)
C10	0.0252 (7)	0.0214 (7)	0.0254 (7)	−0.0026 (6)	−0.0005 (6)	−0.0024 (6)
C11	0.0195 (7)	0.0261 (7)	0.0183 (7)	0.0016 (5)	−0.0025 (5)	−0.0032 (5)
C12	0.0229 (7)	0.0301 (8)	0.0244 (7)	0.0013 (6)	−0.0019 (6)	−0.0071 (6)
C13	0.0330 (9)	0.0462 (10)	0.0281 (8)	−0.0018 (7)	−0.0035 (7)	−0.0164 (7)
C14	0.0440 (10)	0.0676 (12)	0.0175 (7)	−0.0103 (9)	−0.0008 (7)	−0.0124 (8)
C15	0.0427 (10)	0.0492 (10)	0.0215 (8)	−0.0109 (8)	−0.0003 (7)	0.0026 (7)
C16	0.0314 (8)	0.0330 (8)	0.0233 (7)	−0.0061 (6)	−0.0013 (6)	−0.0020 (6)
N1	0.0210 (6)	0.0207 (6)	0.0177 (6)	−0.0009 (4)	−0.0003 (4)	−0.0041 (4)
O1	0.0343 (6)	0.0196 (5)	0.0181 (5)	0.0035 (4)	−0.0033 (4)	−0.0026 (4)
O2	0.0433 (7)	0.0198 (5)	0.0365 (6)	0.0040 (5)	−0.0032 (5)	−0.0055 (5)

### Geometric parameters (Å, º)

**Table:**

C17—O1	1.4312 (16)	C7—C8	1.3706 (19)
C17—H17A	0.98	C8—C9	1.4161 (19)
C17—H17B	0.98	C8—H8	0.95
C17—H17C	0.98	C9—N1	1.3683 (17)
C1—N1	1.3270 (17)	C10—O2	1.2109 (17)
C1—C2	1.4275 (19)	C10—H10	0.95
C1—C11	1.4932 (19)	C11—C12	1.3945 (19)
C2—C3	1.3821 (19)	C11—C16	1.399 (2)
C2—C10	1.4787 (19)	C12—C13	1.383 (2)
C3—C4	1.3998 (19)	C12—H12	0.95
C3—H3	0.95	C13—C14	1.383 (2)
C4—C5	1.4241 (18)	C13—H13	0.95
C4—C9	1.4243 (19)	C14—C15	1.384 (2)
C5—C6	1.3574 (19)	C14—H14	0.95
C5—H5	0.95	C15—C16	1.388 (2)
C6—C7	1.4206 (19)	C15—H15	0.95
C6—H6	0.95	C16—H16	0.95
C7—O1	1.3654 (16)		
			
O1—C17—H17A	109.5	C7—C8—H8	120.2
O1—C17—H17B	109.5	C9—C8—H8	120.2
H17A—C17—H17B	109.5	N1—C9—C8	117.78 (12)
O1—C17—H17C	109.5	N1—C9—C4	122.68 (12)
H17A—C17—H17C	109.5	C8—C9—C4	119.54 (12)
H17B—C17—H17C	109.5	O2—C10—C2	123.68 (14)
N1—C1—C2	122.65 (12)	O2—C10—H10	118.2
N1—C1—C11	114.92 (12)	C2—C10—H10	118.2
C2—C1—C11	122.43 (12)	C12—C11—C16	118.77 (13)
C3—C2—C1	118.67 (12)	C12—C11—C1	119.50 (12)
C3—C2—C10	118.41 (12)	C16—C11—C1	121.69 (12)
C1—C2—C10	122.68 (12)	C13—C12—C11	120.42 (14)
C2—C3—C4	120.13 (12)	C13—C12—H12	119.8
C2—C3—H3	119.9	C11—C12—H12	119.8
C4—C3—H3	119.9	C12—C13—C14	120.48 (15)
C3—C4—C5	123.80 (12)	C12—C13—H13	119.8
C3—C4—C9	117.36 (12)	C14—C13—H13	119.8
C5—C4—C9	118.81 (12)	C13—C14—C15	119.81 (14)
C6—C5—C4	120.86 (12)	C13—C14—H14	120.1
C6—C5—H5	119.6	C15—C14—H14	120.1
C4—C5—H5	119.6	C14—C15—C16	120.15 (15)
C5—C6—C7	119.88 (12)	C14—C15—H15	119.9
C5—C6—H6	120.1	C16—C15—H15	119.9
C7—C6—H6	120.1	C15—C16—C11	120.36 (14)
O1—C7—C8	124.44 (12)	C15—C16—H16	119.8
O1—C7—C6	114.26 (12)	C11—C16—H16	119.8
C8—C7—C6	121.30 (12)	C1—N1—C9	118.45 (11)
C7—C8—C9	119.60 (12)	C7—O1—C17	117.17 (11)
			
N1—C1—C2—C3	2.3 (2)	C3—C2—C10—O2	19.2 (2)
C11—C1—C2—C3	−176.79 (12)	C1—C2—C10—O2	−166.56 (13)
N1—C1—C2—C10	−171.97 (12)	N1—C1—C11—C12	42.75 (18)
C11—C1—C2—C10	9.0 (2)	C2—C1—C11—C12	−138.12 (14)
C1—C2—C3—C4	−0.5 (2)	N1—C1—C11—C16	−135.09 (14)
C10—C2—C3—C4	173.94 (12)	C2—C1—C11—C16	44.04 (19)
C2—C3—C4—C5	−179.58 (12)	C16—C11—C12—C13	0.1 (2)
C2—C3—C4—C9	−1.5 (2)	C1—C11—C12—C13	−177.80 (13)
C3—C4—C5—C6	178.42 (12)	C11—C12—C13—C14	−0.7 (2)
C9—C4—C5—C6	0.4 (2)	C12—C13—C14—C15	1.1 (3)
C4—C5—C6—C7	−0.9 (2)	C13—C14—C15—C16	−0.9 (3)
C5—C6—C7—O1	−178.60 (12)	C14—C15—C16—C11	0.3 (2)
C5—C6—C7—C8	1.0 (2)	C12—C11—C16—C15	0.1 (2)
O1—C7—C8—C9	178.96 (12)	C1—C11—C16—C15	177.95 (14)
C6—C7—C8—C9	−0.6 (2)	C2—C1—N1—C9	−1.7 (2)
C7—C8—C9—N1	179.76 (11)	C11—C1—N1—C9	177.47 (11)
C7—C8—C9—C4	0.1 (2)	C8—C9—N1—C1	179.72 (12)
C3—C4—C9—N1	2.2 (2)	C4—C9—N1—C1	−0.62 (19)
C5—C4—C9—N1	−179.64 (11)	C8—C7—O1—C17	−0.8 (2)
C3—C4—C9—C8	−178.14 (11)	C6—C7—O1—C17	178.80 (11)
C5—C4—C9—C8	0.0 (2)		

### Hydrogen-bond geometry (Å, º)

**Table:**

*D*—H···*A*	*D*—H	H···*A*	*D*···*A*	*D*—H···*A*
C5—H5···O2^i^	0.95	2.48	3.349 (2)	153
C15—H15···O1^ii^	0.95	2.54	3.377 (2)	148

Symmetry codes: (i) −*x*, −*y*+2, −*z*+1; (ii) *x*, *y*+1, *z*−1.
